# Early treatment for children with mental health problems and genetic conditions through a parenting intervention (The GAP): study protocol for a pragmatic randomized controlled trial

**DOI:** 10.1186/s13063-024-08278-4

**Published:** 2024-07-20

**Authors:** Mercedes Serrano, Maria Elias, Marta Llorens, Mercè Bolasell, Helena Vall-Roqué, Laia Villalta

**Affiliations:** 1 Neuropediatric Department, Hospital Sant Joan de Déu, Barcelona, Spain; 2 U-703 Centre for Biomedical Research On Rare Diseases (CIBER-ER), Instituto de Salud Carlos III, Barcelona, Spain; 3 Department of Child and Adolescent Mental Health, Hospital Sant Joan de Déu, Barcelona, Spain; 4 Department of Psychiatry and Forensic Medicine, Universitat Autònoma de Barcelona, Bellaterra, Spain; 5 Department of Genetic and Molecular Medicine, Hospital Sant Joan de Déu, Barcelona, Spain; 6 Child and Adolescent Mental Health Research Group, Institut de Recerca Sant Joan de Déu, Barcelona, Spain

**Keywords:** Development, Genetic syndromes, Early intervention, Mental health, Neurodevelopmental disorders, Parenting, Pragmatic, Randomized controlled trial, Rare disorders, Autism

## Abstract

**Background:**

Children with genetic conditions are at increased risk for mental health and neurodevelopmental problems, often accompanied by significant parental distress. Genetic and family factors can impact children and parents’ mental health. Early parenting interventions, like the Incredible Years® programs, have demonstrated to improve parental distress and children’s mental health. The recent version for young children with language delays or autism spectrum disorder (IY-ASLD®) has shown to be feasible and effective to support parents in their children’s developmental trajectories. The effectiveness of treatments for children with genetic conditions and neurodevelopmental problems is largely unexplored, leaving significant gaps in evidence-based options. Clinicians lack guidance, especially when patients exhibit language or social communication impairments but do not meet diagnostic criteria for a full-blown autism spectrum disorder (ASD). We aim to fill this gap, providing evidence on the feasibility and effectiveness of the IY-ASLD® intervention for such patients.

**Methods:**

We designed a prospective multicenter pragmatic randomized controlled trial including approximately 68 children aged 3 to 7 years, recruited from three tertiary care reference hospitals. Inclusion criteria will necessitate genetic confirmation of a neurodevelopmental disorder along with language, communication, or socialization difficulties. Individuals with an ASD diagnosis will be excluded. All subjects are included in a territorial register for rare conditions (ReMin, Registre de Malalties Minoritàries de Catalunya). Families will randomly be assigned to the intervention or the control group. The intervention will be held online by clinical psychologists and child and adolescent psychiatrists.

**Discussion:**

Our group has recently piloted the online implementation of the IY-ASLD® intervention for the first time in Spain, for parents of children with language delays, socialization difficulties, or ASD, but not genetically determined. Our multicenter research consortium is well-positioned to recruit patients with rare conditions and implement efficient treatment pathways within the National Health System. Given the geographical dispersion of families affected by rare conditions, the online format offers logistical advantages and improved therapy access, enhancing homogeneity across all patients. The results of this study will inform clinicians and policymakers about evidence-based treatment options for this vulnerable and overlooked group of young children.

**Trial registration:**

ClinicalTrials.gov NCT06125093. Date of registration: first submitted 2023–10-23; first posted 2023–11-09. URL of trial registry record.

**Supplementary Information:**

The online version contains supplementary material available at 10.1186/s13063-024-08278-4.

## Background

Children with genetic syndromes commonly present neurodevelopmental problems and mental health comorbidities [1–3]. These difficulties often manifest in early stages, including developmental issues with social communication, emotion, and behavior regulation. The persistence of these symptoms can negatively affect children’s developmental trajectories, including the emergence of social dysfunction, comorbid mental health disorders, and future maladaptation [4]. Despite the complex phenotypic presentations of children with genetic syndromes, and the high prevalence of impairing symptoms, they have not been thoroughly studied from a developmental perspective. Additionally, parents of children with early neurodevelopmental problems often report elevated distress levels and mental health difficulties, which may, in turn, potentially affect their children’s mental health [5, 6].

Thus, early intervention is crucial to improve these outcomes in children with neurodevelopmental problems. Early signs of concern, such as communication, socialization, or dysregulation problems, should be targeted and treated, with no need to wait for a full-blown diagnosis. This dimensional approach allows treatment to reach young children with a broad spectrum of neurodevelopmental difficulties, rather than being limited to particular diagnostic categories. In this regard, young children with genetic conditions and mental health problems represent a clinical and research priority. Firstly, children with genetic syndromes have complex heterogeneous clinical presentations, both within and between genetic conditions. Their atypical symptoms rarely meet full criteria for mental health disorders categorically classified in the Diagnostic and Statistical Manual of Mental Disorders (DSM), despite impairing children’s functioning [7]. Consequently, their mental health problems are typically overlooked, and early treatment opportunities are missed. This can worsen their development and mental health course and future prognosis. Secondly, generating knowledge in this group of children is challenging due to the rarity of their conditions, making it difficult to achieve a critical mass and statistical soundness. Finally, these patients are geographically dispersed, posing a challenge in ensuring equitable access to intensive evidence-based treatments [8].

In view of such particularities, the assistance of these patients requires a flexible health system, a dimensional mental health perspective, and a collaborative scientific approach in order to efficiently diagnose and treat their impairing mental health symptoms. Their care pathways need to be implemented in early childhood, following standardized interventions for homogeneous groups of patients, and led by experienced clinicians. These specific conditions can only be effectively managed by highly specialized multidisciplinary teams with extensive knowledge of their unique characteristics, providing assistance to a large patient population.

Young children with genetic conditions present particular behavioral phenotypes (i.e., a characteristic pattern of cognitive, linguistic, social, and motor abnormalities), which commonly overlap with recognized diagnosis such as autism spectrum disorder (ASD). Despite the high prevalence of ASD in children with genetic syndromes (greater than 50% in Sotos syndrome or duplication 15q11q13, but also relevant in Prader-Willi syndrome (13%) and Williams syndrome (12%), among others) [1–3, 9, 10], a growing body of evidence suggests that these individuals might have an atypical profile of ASD phenomenology, distinct from idiopathic ASD [2]. More than 21 genetic syndromes have been described to present autistic-like behaviors, such as social deficits and repetitive behaviors, which might not meet full criteria for an ASD diagnosis [11–16].

Unfortunately, these patients are less likely to access interventions targeting their impairing symptoms, as there is a lack of evidence regarding effective treatment options, and only the most severe cases with prominent symptoms in all domains are referred to ASD care pathways.

Early parenting interventions have increasingly shown a large and sustained effect on children neurodevelopmental and mental health outcomes [17–19]. Group interventions show promise, at a relatively low-cost, as a valuable resource to help parents cope with children’s behavioral, social, and emotional difficulties. This therapeutic approach has demonstrated effectiveness in improving dysfunctional parenting styles, reducing children’s behavioral problems, and increasing parents’ ability to facilitate their children’s communication skills and vocabulary [20]. Group interventions also provide social support for parents. This is especially important for parents of children with genetic syndromes, who present high stress levels as well as a lack of structured guidance on effective ways to promote their children’s development and mental health [5, 6].

The Incredible Years® (IY®) parenting programs [21] are a set of interventions recommended by the NICE guidelines to improve parenting skills and children’s behavioral problems [22]. The effectiveness of the IY® parenting programs has been widely demonstrated in multiple randomized controlled trials, showing an improvement in parental stress levels, depression, and parental coping, as well as in children’s behavior difficulties [23, 24]. Specifically, the IY® program has been adapted to target the needs and concerns of parents of young children in the autism spectrum or with language delays (IY-ASLD®) [25]. A pragmatic randomized controlled trial has supported the feasibility and acceptance of delivering this intervention for ASD children in the UK National Health Service [26]. Within the Spanish mental health system, this program has recently been piloted for parents of children with ASD and preterm children with communication or socialization difficulties [27], showing high satisfaction and compliance with the intervention [28]. However, this intervention has never been implemented for children with neurodevelopmental problems related to genetic syndromes, a particularly vulnerable and hard-to-ascertain sample.

## Objectives

The primary objective of this study is to examine the feasibility and preliminary effectiveness of the IY-ASLD® intervention for children with genetic syndromes and mental health problems. Specifically, we aim to evaluate the parental and clinician compliance and satisfaction with the intervention.

Additionally, we seek to gather preliminary evidence on its effectiveness in reducing parental distress, enhancing parenting skills, improving parent–child relationships, and, secondarily, addressing overall children’s mental health problems.

To achieve these goals, we designed a randomized controlled trial that will, for the first time, overcome challenges associated with the limited availability of knowledge and its geographical dispersion when treating children with rare genetic syndromes.

## Methods

### Design and setting

We will conduct a randomized, controlled, parallel-group, exploratory trial within the Spanish Public Health System, where participating families will be randomly allocated to the intervention group (receiving the IY-ASLD® program) or to the treatment as usual (TAU) group on a 1:1 ratio.

The GAP project will be conducted from March 2023 to September 2024 in three tertiary care hospitals in the province of Barcelona (Sant Joan de Déu Hospital (SJDH), Vall d’Hebron Hospital (VHH), and Parc Taulí Hospital (PTH)) conforming together a territorial expertise network (XUEC-MCC: Xarxa d’Unitats d’expertesa clínica-malalties minoritàries cognitiu conductuals). This network was created and accredited in 2015 to guarantee equal and full access to health and research opportunities regardless of geographic location for children with genetic syndromes that are included in the rare conditions register (ReMin, Registre de Malalties Minoritàries de Catalunya). This is the most exhaustive national registry and is custodied by the National Health System (CatSalut). This registry provides access to a sample that is difficult to ascertain, thereby overcoming the rarity of genetic conditions. The three participating hospitals have multidisciplinary units led by a patient-centered policy that can go beyond institutional or geographical limits.

### Eligibility criteria

Participating families will be recruited from the three units within each hospital, and patients will fulfill the following criteria:

1. Children aged 3.0–7.11 years at recruitment
2. Children with genetically determined neurodevelopmental conditions (see Supplementary File 1)
3. For children aged up to 5.11 years:
  Withdrawn problems (defined as Child Behavior Checklist (CBCL/1.5–5) scores above the borderline clinical range for the “Withdrawn” domain, T-score ≥ 65)
    AND/OR
  Pervasive neurodevelopmental problems (defined as CBCL/1.5–5 scores above the borderline clinical range for the “Pervasive developmental problems” domain, T-score ≥ 65)
    AND/OR
  Socialization problems (defined as Vineland-III scores below 1SD in the socialization subdomains)
4. For children aged over 6.0 years:
  Social problems (defined as CBCL/6–18 scores above the borderline clinical range for the “Social problems” domain, T-score ≥ 65)
    AND/OR
  Thought problems (defined as CBCL/6–18 scores above the borderline clinical range for the “Though problems” domain, T-score ≥ 65)
    AND/OR
  Socialization problems (defined as Vineland-III scores below 1SD in the socialization subdomains)
5. Parents/caregivers showing good understanding of Spanish or Catalan language
6. Parents/caregivers consenting to take part in the study and signing the informed consent

The following are the exclusion criteria:

1. Children with a previously known diagnosis of ASD
2. Children scoring above the diagnostic cut-off for ASD in the Autism Diagnostic Observation Schedule (ADOS-2) assessment
3. Parents/caregivers attending another structured parenting program
4. Children in the care of their local authority

### Recruitment and sample size calculation

**Fig. 1:**
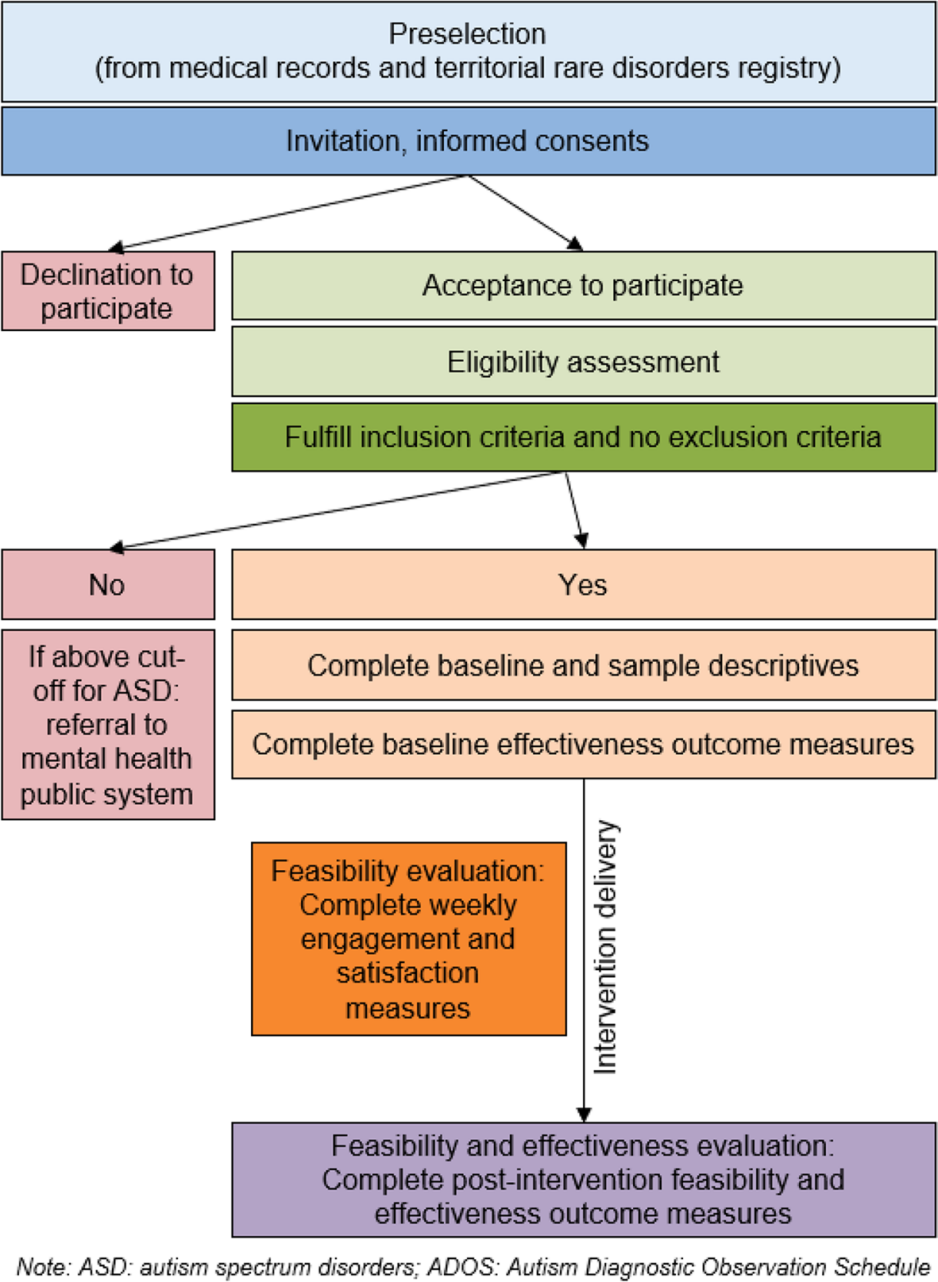
Recruitment and evaluation process that will be followed

Parents will be recruited from the specialist units in genetic neurodevelopmental syndromes at the three hospitals. Figure 1 shows the recruitment process that will be followed. Clinicians will present the study to preselected eligible families and will ask them for permission to be contacted by the research assistant, who will then explain the study further. If the family is interested, an appointment to evaluate all the inclusion criteria will be set up and the parents will be asked to sign the informed consent and to complete the pre-intervention assessment (see “Eligibility criteria” section). Any children excluded from the study at that point due to scoring above diagnostic cut-off for ASD at the ADOS-2 will be referred for a specialized mental health assessment within the public system. Participants will be able to discontinue the treatment sessions or drop out from the control group at any point at their request. The participation or disengagement from the study will not affect their usual treatment.

The sample size was primarily based on the recommendations for feasibility studies [29, 30], which suggest sample sizes of between 24 and 55 participants, and on similar previous studies [26, 31]. Secondarily, we did a sample size calculation based on the main secondary outcome, parental stress, measured through the Parental Stress Index Short Form (PSI-SF) questionnaire [32], as previous studies [31] have used PSI to estimate sample sizes. This is a 36-item scale, with a range of 180 points. There is data showing that a decrease of 16.5 points in the total scale stress score can be seen after attending an IY program [33]. Given that this study will be conducted with parents of children presenting neurodevelopmental difficulties, we anticipated that the decrease in the PSI-SF score would be lower. We estimated the necessary sample size considering a power of 80%, *α* = 0.05, a difference between pre- and post-test of 10 points, and a standard deviation of 20, using a paired-samples *t*-test. We estimated 34 participants needed per arm of the study. Thus, we aim to recruit approximately 68 participants. The dropout rate has not been included when calculating the sample size, considering that a sample of 68 participants is similar than previous studies [26, 31] and in line with recommendations by the National Institute of Health Research [29]. Using the public registry for rare diseases (ReMin: Registre de Malalties Minoritàries) we have identified a minimum number of 184 patients within the age interval and confirmed genetic syndrome (see inclusion criteria above).

Regarding the sample size for the individual interviews that will be conducted with parents after the intervention, a minimum of two parents of each intervention group will be interviewed. We will aim for a representative sample in terms of children’s diagnosis, age, and gender. Parents who drop out from the intervention will also be invited to be interviewed. Data saturation will be used to determine the final sample size.

For the post-intervention individual interviews with clinicians, all clinicians who delivered the intervention will be interviewed after the intervention phase.

### Randomization

Randomization (intervention or TAU condition on a 1:1 ratio) will take place once all the participating families have completed baseline measures. Once included in the trial, the randomization code will be generated centrally by a statistician using PROC PLAN available in the SAS® version 9.4 statistical program. One list will be generated with blocks. The seed number will be chosen randomly using RANUNI (function available in SAS), because random numbers are known to follow a uniform distribution. Allocation will be stratified by cognition developmental level depending on the final sample characteristics (Developmental Profile-3 (DP-3) cognition scale below or above 70). An independent researcher will carry out the randomization process, and researchers responsible for recruitment or intervention delivery will not be involved. Due to the pragmatic and clinically focused nature of the study, further blinding procedures will not be possible.

### Intervention

Clinical psychologists (ME, LM, IS, BM) and child psychiatrists (LV, MLL, GE, SP) working in specialized mental health services for neurodevelopmental disorders within the three hospitals will deliver the intervention.

The IY-ASLD® program [25] encourages positive parent–child relationships to promote children’s social competence, peer relationships, language skills, pre-academic coaching, and emotion and behavior regulation. The intervention encourages parents to play in a child-directed way but with a specific focus on enhancing children’s communication and social engagement. It also focuses on how to use positive discipline to set limits and handle misbehavior. During the coronavirus disease-19 (COVID-19) pandemic, the IY-ASLD® program developers adapted the intervention to be performed online (22 sessions approximately). Each group includes a maximum of 8–10 participants, although the online format recommends keeping the number of participants in the lower range, particularly if children have complex clinical presentations. The intervention includes video modeling, role-playing, and discussion activities with parents and emphasizes the importance of practice-based learning in the sessions and at home. Families will be contacted weekly between sessions to encourage home-based practice.

Fidelity to the treatment manual will be ensured in different ways. The intervention will be conducted by experienced psychiatrists and clinical psychologists, officially trained in the IY-ASLD® model and its online adaptation by an accredited trainer. Group leaders will complete weekly fidelity checklists and will receive group supervision by a certified supervisor in two different time points throughout the intervention. Due to the pragmatic nature of the study, further supervision or accreditation processes will not be possible. The TAU condition involves outpatient appointments with neuropediatricians to monitor children’s developmental level and drug prescription when needed. All children under 5 years of age are also treated in Early Childhood Developmental Centers based in the community, where they receive individual unstructured interventions to foster children’s development. Children aged over 5 years might or might not receive psychological or psychiatric treatment in specialized child and adolescent mental health community centers. Families allocated to the intervention group will also receive TAU.

### Measures

Table 1 summarizes the study timeline and the measures that will be used.

**Table 1:** Overview of the study timeline and measures

**Time points**	**Study period**				
	**Enrollment**	**Allocation**	**Weekly intervention (22 sessions)**		**Close-out**
	**March to November 2023**	**November 2023**	**November 2023** **Session 1**	**June 2024** **Session 22**	**June to September 2024**
**Enrollment**					
Eligibility screen	X				
Informed consent	X				
Randomization and allocation		X			
**Intervention**					
IY-ASLD® group	X	X	X	X	X
TAU group	X	X			X
**Assessments**					
***Baseline variables***					
Sociodemographic variables: Hollingdale’s Index of Social Position	X				
Social Communication Questionnaire (SCQ)	X				
Autism Diagnostic Observation Schedule-2 (ADOS-2)	X				
Vineland Adaptive Behavior Scale-III (VABS-III)	X				
Developmental Profile-3 (DP-3)	X				
Child Behavior Checklist (CBCL)	X				X
***Outcome variables and other measures***					
Autism Program Parent Weekly Evaluation			X	X	
Autism Program Parent Final Satisfaction Questionnaire				X	X
Individual interviews with parents					X
Individual interviews with clinicians					X
Parent Stress Inventory-Short Form (PSI-SF)	X				X
Beck Depression Inventory (BDI)	X				X
Alabama Parenting Questionnaire-Preschool version (APQ-Pr)	X				X
Autism-Specific Five Minute Speech Sample (ASFMSS)	X				X
Child Behavior Checklist (CBCL)	X				X

*IY-ASLD*® Incredible Years Autism Spectrum and Language Delays program®, *TAU* treatment as usual

#### Baseline and sample descriptors

At baseline, sociodemographic and children clinical variables will be collected from clinical notes and parent reports. Socioeconomic status will be determined using Hollingdale’s Index of Social Position [34].

The following questionnaires, which have already shown valid and reliable results in children with neurodevelopmental problems, will be administered before the intervention:

- Social Communication Questionnaire (SCQ) [35]: this screening tool is a 40-item parent report measure, with a yes/no format, based on the Autism Diagnostic Interview-Revised (ADI-R) [36]. The Lifetime version of the questionnaire will be applied to all children, from 3 to 7 years old. Children are likely to be on the autism spectrum when their total scores are 8 or higher.
- Autism Diagnostic Observation Schedule (ADOS-2) [37]: it is a semi-structured assessment of social interaction, communication, and play exposing the child to situations that elicit spontaneous behaviors in standardized contexts. Modules 1 to 3 will be selected in accordance to the children’s language level. To receive an autism spectrum diagnosis by the ADOS-2 algorithm, an individual’s score must exceed a total cut-off score.
- Vineland Adaptive Behavior Scale-III (VABS-III, parent/caregiver report form) [38]: it assesses the adaptive functioning of the child in the family environment. It is composed of the following domains: communication, socialization, daily life skills, and motor skills. It also generates an index of adaptive behavior.
- Developmental Profile-3 (DP-3) [39]: this is a 180-item parental questionnaire assessing developmental delays in different domains: motor, adaptative, socio-emotional, cognitive, and communication. It also computes an overall general development score.
- Child Behavior Checklist (CBCL 1.5–5 and 6–18) [40, 41]: this questionnaire is a component of the Achenbach System of Empirically Based Assessment (ASEBA). It is a parent-reported 99-item (CBCL 1.5–5) and 113-item (CBCL 6–18) inventory that measures adaptive functioning and behavioral, emotional, and social problems. It is made up of eight syndrome scales, which are grouped into two main categories: internalizing and externalizing. The sum of scores forms a total problem score, and it also includes subdomains. This questionnaire will also be administered after the intervention as an exploratory measure.

#### Outcome measures

##### Primary outcomes (feasibility)

- Parents’ engagement with the program and participant retention will be monitored weekly throughout the intervention using an attendance sheet, expecting they will attend at least 15/22 sessions with a minimum of 50% of parents finishing the program.
- Autism Program Parent Weekly Evaluation [42]: this instrument is part of the IY-ASLD® program materials and will be administered weekly throughout the intervention to collect information regarding parents’ compliance and satisfaction.
- Autism Program Parent Final Satisfaction Questionnaire [42]: this questionnaire is included within the IY-ASLD® program. It will be used to measure the acceptability and satisfaction with the intervention after the last session.
- Parents’ overall experiences with the program will be explored by means of individual interviews. These interviews will be conducted after the last session to explore from a qualitative perspective: (1) parents’ acceptability, satisfaction, and overall experience with the intervention and (2) parents’ perceived changes in their parenting skills and parental distress after the intervention.
- Clinicians’ experiences with the intervention will also be qualitatively assessed by means of individual interviews with all clinicians who delivered the intervention. These interviews will be conducted after the last session.

##### Secondary outcomes (effectiveness for parental outcomes)

All the following questionnaires will be administered before and after the intervention:

- Parent Stress Inventory-Short Form (PSI-SF) [32, 43]: 36-item questionnaire to assess parental stress associated with the care of their offspring. It is aimed at parents of children from 1 month to 12 years of age. It has three domains: parental distress, parent–child dysfunctional interaction, and difficult child, which combine to form a total stress scale.
- Beck Depression Inventory (BDI) [44]: 21-item screening tool assessing the severity of depressive symptoms. It is a standardized and validated questionnaire, often used in adults’ mood disorder assessments.
- Alabama Parenting Questionnaire-Preschool revision (APQ-Pr) [45]: 32-item parent-reported questionnaire measuring parenting practices associated with disruptive child behaviors. This version has 3 dimensions: positive parenting, inconsistent parenting, and punitive parenting.
- Autism-Specific Five Minute Speech Sample (ASFMSS) [46]: this is a narrative 5-min interview used to measure parental expressed emotions for children with ASD and related disorders. Parents are asked to speak about their child and the parent–child relationship. Speech samples are audiotaped, transcribed, and coded following six global categories: (a) initial statement, (b) warmth, (c) relationship, (d) emotional over-involvement, (e) critical comments, and (f) positive comments. This tool has been translated and validated in Spanish families [47].

##### Other exploratory measures

Preliminary effectiveness of the intervention on children’s problems will be explored with the Child Behavior Checklist (CBCL explained above).

### Data collection and data analysis

#### Quantitative data collection and analysis

Data will be collected at baseline (before performing randomization) and after finishing the IY-ASLD® intervention. Parents consenting to participate will be offered a hospital appointment with a research assistant to evaluate children inclusion criteria (children questionnaires and ADOS-2). If the child meets inclusion criteria, parents will be contacted on the phone to complete the remaining questionnaires (including all children descriptors, effectiveness parental outcome measures, and the ASFMFSS voice recording). If both parents agree to participate in the study, they will fill out children measures together by consensus and parental outcome measures individually.

Descriptive analyses will be used for recruitment, attrition, and satisfaction rates of the participants with the program. Differences in baseline and descriptive variables between intervention and TAU conditions will be described and controlled in the main analyses. For effectiveness outcomes, the analysis will follow an intention-to-treat basis. Relevant confounding variables, such as sociodemographic variables, developmental level, or treatment site, will be added as covariates. Statistical analysis will be performed using SPSS (IBM Corp., Armonk, NY). The Wilcoxon test will be used to compare quantitative paired variables between the different evaluations through time. Chi-square test will be used to compare categorical variables with effectiveness variables. Spearman’s rank correlation coefficient test will be applied to study the relationship between the quantitative variables (such as clinical scores). We will also estimate 95% confidence intervals and effect sizes.

All statistical analyses will be two-sided and *p* values < 0.05 will be considered significant.

Imputation models will be used to handle missing data.

#### Qualitative data collection and analysis

Interviews with parents and clinicians will take place after finishing the intervention. They will be conducted online by a psychologist trained in qualitative methods and will be audio-recorded and transcribed verbatim.

A qualitative thematic analysis will be performed to analyze the data. All transcripts will be coded, analyzed, and discussed by at least two independent researchers not involved in the intervention delivery. The coding process will be inductive: themes and associated codes will be generated from the interviews data. The coding process will be supported by the software Atlas.ti.

### Ethical issues

Signed informed consent will be obtained from the parents/caregivers of study participants. The study protocol and consents will be reviewed and approved by the ethics committee of each participating site: Research & Ethics Committee of SJDH, Clinical Research Ethics Committee (CEIm) of VHH, and Committee for Ethical Clinical Investigation of PTH. A copy of the consent will be given to the patient’s parent/caregiver. All data will be anonymized and stored by the research group. The study will be conducted in accordance with the Declaration of Helsinki, Good Clinical Practices, and applicable regulatory requirements. Our data management plan will consider FAIR principles (Findable, Accessible, Interoperable, and Re-usable). Also, as it is a research project on rare diseases, it will also consider the principles established by the International Rare Diseases Research Consortium.

Any modifications to the protocol which may impact on the conduct of the study, potential benefit of the patient or may affect patient safety, including changes of study objectives, study design, patient population, sample sizes, study procedures, or significant administrative aspects will require a formal amendment to the protocol. Such amendment will be agreed upon by the research group and approved by the Ethics Committee prior to implementation. It will also be registered at ClinicalTrials.gov.

As The GAP involves a low-risk intervention, a data monitoring committee was not required by the competent ethics committees, and there are no anticipated problems detrimental to participants. Any adverse events and other unintended effects will be reported to the ethics committees. Following their advice, mitigation plans will be implemented accordingly before the next intervention session.

The project management group will meet bimonthly to review trial conduct and will be in close contact with the Ethics Committees. This process will be independent from investigators and the sponsor.

We used the SPIRIT checklist when writing our report [48] (see Supplementary File 2).

## Dissemination plans

The results of the study, regardless of whether they are in favor of the intervention or TAU group, or inconclusive, will be published in a peer-reviewed journal. Individuals with rare conditions can often be identified by their unique molecular findings, making it challenging to share data without revealing their identity. However, open-access publication with exhaustive data in supplementary files is part of our plan. Additionally, preprint open-access websites will be used once the results are ready to be submitted for publication. The investigators will also communicate the trial results to the national and international scientific community and healthcare providers at different conferences. The results will also be shared through social media channels of the participating hospitals and sponsor, as well as through the Centro de Investigación Biomédica en Red Enfermedades Raras (CIBERER) and Fundació de Recerca i Docència Sant Joan de Déu’s websites. A lay summary will be shared with all project participants and with rare conditions’ patients’ and families’ associations. The trial registry will be updated.

We do not intend to use professional writers for our publications. Authorship eligibility will be based on the recommendations from the International Committees of Medical Journal Editors.

## Discussion

To our knowledge, there is no existing evidence on group-based parenting programs for children with genetic syndromes and early neurodevelopmental problems. The results of the current study would provide novel evidence on the feasibility and effectiveness of the IY-ASLD® intervention implemented in existing services. This would also inform policy makers on new and efficient care pathways for such vulnerable and overlooked group of patients.

The implementation of this intervention has been piloted in the National Health Service in the UK. A pragmatic, feasibility randomized controlled trial was done with 29 parents treated with 12 weekly face-to-face sessions and 29 parents in a TAU control group. Results showed high acceptability, fidelity, and satisfaction with the program [26]. The IY-ASLD® has also been piloted within the Spanish Mental Health System. A recent multicentric randomized pilot study was conducted by researchers of the present investigation [27]. The intervention was conducted during the COVID-19 pandemic and 22 weekly sessions were carried out online. Preliminary results also showed high levels of satisfaction and adherence to the program in parents of young children with language delay, socialization difficulties, or ASD [28]. This suggests that it is feasible to implement the online intervention reliably in our Public Mental Health System.

Neurodevelopmental disorders of genetic basis are rare conditions. Their complex multiorganic implications lead to particular clinical presentations, with commonly underreported associated mental health difficulties [9]. This study will generate novel scientific data on the phenotype description of early mental health problems of young children with genetic syndromes, as well as on treatment options for such difficulties. Thus, The GAP study will provide new scientific data while setting up initial guidelines to implement standards of mental health treatment in the public mental health system.

This scientific gap is also worrying from a societal point of view. Families whose infants are diagnosed with a genetic condition face high stress levels related to parenting children with behavior, communication, and social difficulties, and in relation to how the diagnosis will affect their children’s mental health [49]. In this regard, parents tend to look for peer-to-peer support in family associations. The GAP project will bring families with similar problems closer, creating opportunities for peer-to-peer support while providing rigorous and specialized treatment options to improve parent and children mental health.

The project will represent a step forward in generating homogeneous treatment pathways within the different specialized genetic and mental health units that assist these patients in our health system.

Also, as the intervention will be carried out online, all families will have the opportunity to access intense evidence-based treatments regardless of their area of residence. Due to the geographical breakdown of families living with rare conditions, several advantages of the online format could be highlighted, such as better family logistics and improved access to therapy. Conducting this intervention in an online format is a challenge for clinicians, but determining its feasibility will inform policy makers of new treatment options to be implemented in our territory. It is also of note that support groups are an important resource for many families living with children with genetic syndromes, and meeting via teleconference has shown to be a facilitating factor [50].

Furthermore, we will combine quantitative and qualitative methods, and this will be helpful to better understand the outcomes of the study. Mixed methods research is increasingly recognized as valuable, because it can potentially draw upon the strengths and perspectives of quantitative and qualitative approaches respectively [51]. As O’Cathain et al. [52] conclude, embedding qualitative methods to clinical trials can be of help in improving the external validity of trials or facilitating interpretation of the trial findings, among other aspects.

As a limitation, importantly, there is great heterogeneity among different genetically determined neurodevelopmental disorders and, within their rarity, some of them will be overrepresented in the sample due to greater prevalence. Moreover, it is unfeasible to have representation of all genetic syndromes. Therefore, we may need to assume that the results can be extrapolated to other genetic syndromes that present with communication or ASD-like symptoms. On the contrary, as a benefit of the overrepresentation of certain syndromes, the presence of a consistent group of individuals with the same adaptive behavioral phenotype divided between the control and the intervention group will help to validate the results, particularly for that given phenotype, regardless of the neurodevelopmental genetic condition.

Another potential limitation lies within the fact that the IY-ASLD® program is addressed at families of children with ASD or language delays. Considering the heterogeneity of syndromes that will be present at each intervention group, some specific contents of the intervention might not be relevant to some families. To mitigate this limitation, groups will be created based on children’s clinical profile (developmental and language level), with the aim that they are as homogeneous as possible. This will allow group leaders to focus in greater detail on the areas more relevant to each group of parents.

Furthermore, despite the above mentioned advantages of the online format, this might entangle its own limitations as well [53], which will need to be considered carefully.

In summary, this study will provide with new evidence to guide future psychological treatments for children living with genetic conditions and neurodevelopmental problems.

## Trial status

The current protocol is version 1 dated April 21, 2024. The protocol for the current study was registered in ClinicalTrials.gov (ID number: NCT06125093) in October 2023. The study began in March 2023, and the anticipated finish date is September 2024. The recruitment process was carried out from March 2023 to November 2023, and the intervention is currently being delivered and expected to finish in June 2024. We were unable to submit this manuscript early due to time constraints: however, it was registered in ClinicalTrials.gov before the recruitment was completed.

### Supplementary Information

Additional file 1. Neurodevelopmental and mental health problems associated with the genetic syndromes included in the study.

Additional file 2. SPIRIT checklist.

## Data Availability

All relevant data and methods are reported in the article and in the supplementary material. The full protocol, the final de-identified dataset, and statistical code will be made available after publication of the study outcomes, if required by the journal policy or via a request to the corresponding author.

## Abbreviations

ADOS-2: Autism Diagnostic Observation Schedule-2
APQ-Pr: Alabama Parenting Questionnaire-Preschool revision
ASD: Autism spectrum disorder
ASFMSS: Autism-Specific Five Minute Speech Sample
BDI: Beck Depression Inventory
CBCL: Child Behavior Checklist
CEIm: Clinical Research Ethics Committee
COVID-19: Coronavirus disease-19
DP-3: Developmental Profile-3
DSM: Diagnostic and Statistical Manual of Mental Disorders
IY-ASLD®: Incredible Years-Autism Spectrum and Language Delay
PSI-SF: Parent Stress Inventory-Short Form
PTH: Parc Taulí Hospital
ReMin: Registre de Malalties Minoritàries de Catalunya
SCQ: Social Communication Questionnaire
SJDH: Sant Joan de Déu Hospital
TAU: Treatment as usual
VABS-III: Vineland Adaptive Behavior Scale-III
VHH: Vall d’Hebron Hospital
XUEC-MCC: Xarxa d’Unitats d’expertesa clínica-malalties minoritàries cognitiu conductuals
