# Supplementary material for: Early treatment for children with mental health problems and genetic conditions through a parenting intervention (The GAP): study protocol for a pragmatic randomized controlled trial
Source: Trials. 2024 Jul 20;25:496. doi: 10.1186/s13063-024-08278-4 (PMC11265099; doi:10.1186/s13063-024-08278-4)
Supplement: Supplementary file 1 — Additional file 1. Neurodevelopmental and mental health problems associated with the genetic syndromes included in the study. [file 13063_2024_8278_MOESM1_ESM.docx]

**Supplementary File 1**

**Neurodevelopmental and mental health problems associated with the genetic syndromes included in the study.**

| **Syndrome** | **Neurodevelopmental problems** |
| --- | --- |
| 47,XYY syndrome | Variable IDD, ASD, ADHD, impulsivity, poor adaptation to social situations, behavioral problems related to externalizing behaviors. [1] |
| 1q21 deletion syndrome | Variable IDD, learning disabilities, speech delay, risk of schizophrenia. [2] |
| 1q21 duplication syndrome | Variable IDD, ASD, ADHD, impaired communication and socialization skills. [2, 3] |
| 1p36 deletion syndrome* | IDD, ADHD, frequent ASD traits, temper tantrums, self-aggression, variable language development impairment. [4, 5] |
| 3q29 microdeletion syndrome | Pervasive developmental disorder not otherwise specified, anxiety, risk of schizophrenia. [6, 7] |
| Cri-du-chat syndrome (5p minus) | Variable IDD, ASD, impulsivity and overactivity, excessive positive affect (mood, interest, pleasure). Limited verbal skills: simple language implemented with alternative communication. Strength in receptive compared to expressive language. [8 – 10] |
| Smith Magenis syndrome | IDD, ADHD, strong desire for social interaction. Maladaptive behaviors such as self-injury, contrasting with challenging behavior, hyperactivity, stereotypical behaviors, impulsivity and overactivity. [11]  Autistic-like behaviors. [12] |
| Williams syndrome (7q11.21 microdeletion) | Variable IDD, ASD, difficulties making friends but overfriendly, maladaptive behaviors, non-social anxiety, difficulties in social reciprocity. [11, 13] |
| Prader Willi syndrome | Variable IDD, ASD, solitary behavior, social withdrawal, poor peer relations, pronounced repetitive and compulsive behaviors, difficulties with routine changes. [11, 14, 15] |
| 15q11.2 microdeletion syndrome | IDD, ASD, ADHD, OCD, self-injurious behavior, oppositional defiant disorder, schizophrenia, paranoid psychosis. [16] |
| 15q11q13 duplication syndrome, Idic15* | IDD, a great majority fulfil the diagnostic criteria for ASD. Expressive language normally severely impaired, frequent echolalia. Communicative intention is frequently absent. [17] |
| 22q11.2 microdeletion syndrome | Variable IDD, ASD, difficulties to maintain relationships, social domain and expressive language delays, high risk of development of psychotic disorders. [13] |
| CHARGE syndrome | Variable IDD, ASD. [13]  Very goal directed and persistent with sense of humor, socially interested but immature, repetitive behaviors, high degree of sensation seeking, difficulties in self-regulation, difficulty with shifting attention; easily lost in own thoughts. [18, 19] |
| Coffin-Siris syndrome | ASD, deficits in social communicative skills, repetitive and/or stereotyped behaviors, reciprocal social interaction, delayed speech development. [20, 21] |
| Fragile X syndrome | IDD, ASD (22%), ADHD, sensory hypersensitivity, stereotypical behavior, hand flapping, echolalia, language delay, social and communicative impairment, repetitive behavior. [10, 13, 22] |
| KBG syndrome | IDD, ASD, ADHD, anxiety, aggressive or compulsive behavior. [23 – 25] |
| Kleefstra syndrome | IDD, ADHD, ASD very variable among individuals but present in more than 60%, stereotypies, mild self-injurious behaviors. [26 – 28] |
| Noonan syndrome | Variable IDD, ASD (15%), suboptimal organization skills and compromised abilities to structure complex information. There is an important correlation with the underlying affected gene. [29] |
| Lowe syndrome (OCRL)* | Variable IDD, ADHD, temper tantrums, excessive positive affect (mood, interest, pleasure), autistic traits are frequent with stereotypes, restricted interests, lack of cognitive flexibility. [10, 30] |
| Sotos syndrome | Variable IDD, ASD, ADHD, difficulties with peer group relationships, lack of awareness of social cues, speech delays. [31] |

Note: ADHD: attention deficit hyperactivity disorder; ASD: Autism spectrum disorder; IDD: Intellectual developmental disorders; OCD: Obsessive compulsive disorder; OCRL: Oculocerebrorenal syndrome of Lowe.

Variable IDD: may reflect individuals with cognitive functions in the low range of normality.

*The presence of early-onset epilepsy or refractory epilepsy in this condition highly determines the severity of the communication and behavioural impairment.

**References**

[1] Ross JL, Roeltgen DP, Kushner H, Zinn AR, Reiss A, Bardsley MZ, McCauley E, Tartaglia N. Behavioral and social phenotypes in boys with 47,XYY syndrome or 47,XXY Klinefelter syndrome. Pediatrics. 2012;129(4):769-78. doi: 10.1542/peds.2011-0719.

[2] Brunetti-Pierri N, Berg JS, Scaglia F, Belmont J, Bacino CA, Sahoo T, Lalani SR, Graham B, Lee B, Shinawi M, Shen J, Kang SH, Pursley A, Lotze T, Kennedy G, Lansky-Shafer S, Weaver C, Roeder ER, Grebe TA, Arnold GL, Hutchison T, Reimschisel T, Amato S, Geragthy MT, Innis JW, Obersztyn E, Nowakowska B, Rosengren SS, Bader PI, Grange DK, Naqvi S, Garnica AD, Bernes SM, Fong CT, Summers A, Walters WD, Lupski JR, Stankiewicz P, Cheung SW, Patel A. Recurrent reciprocal 1q21.1 deletions and duplications associated with microcephaly or macrocephaly and developmental and behavioral abnormalities. Nat Genet. 2008;40(12):1466-71. doi: 10.1038/ng.279.

[3] National Institutes of Health [Internet]. Chromosome 1q21.1 duplication syndrome. Available from: <https://www.ncbi.nlm.nih.gov/medgen/382715> Accessed 2 April 2024.

[4] Briegel W. Psychiatric Comorbidities in 1p36 Deletion Syndrome and Their Treatment-A Case Report. *Int J Environ Res Public Health*. 2021;18(22):12064. doi: 10.3390/ijerph182212064

[5] National Institutes of Health [Internet]. Chromosome 1p36 deletion syndrome. Available from: <https://www.ncbi.nlm.nih.gov/medgen/334629> Accessed 2 April 2024.

[6] Città S, Buono S, Greco D, Barone C, Alfei E, Bulgheroni S, Usilla A, Pantaleoni C, Romano C. 3q29 microdeletion syndrome: Cognitive and behavioral phenotype in four patients. Am J Med Genet A. 2013;161A(12):3018-22. doi: 10.1002/ajmg.a.36142.

[7] Murphy MM, Lindsey Burrell T, Cubells JF, España RA, Gambello MJ, Goines KCB, Klaiman C, Li L, Novacek DM, Papetti A, Sanchez Russo RL, Saulnier CA, Shultz S, Walker E, Mulle JG. Study protocol for The Emory 3q29 Project: evaluation of neurodevelopmental, psychiatric, and medical symptoms in 3q29 deletion syndrome. BMC Psychiatry. 2018;18(1):183. doi: 10.1186/s12888-018-1760-5.

[8] Cerruti Mainardi P. Cri du Chat syndrome. Orphanet J Rare Dis. 2006;1:33. doi: 10.1186/1750-1172-1-33.

[9] Moss JF, Oliver C, Berg K, Kaur G, Jephcott L, Cornish K. Prevalence of autism spectrum phenomenology in Cornelia de Lange and Cri du Chat syndromes. Am J Ment Retard. 2008;113(4):278-91. doi: 10.1352/0895-8017(2008)113[278:POASPI]2.0.CO;2.

[10] Oliver C, Berg K, Moss J, Arron K, Burbidge C. Delineation of behavioral phenotypes in genetic syndromes: characteristics of autism spectrum disorder, affect and hyperactivity. J Autism Dev Disord. 2011;41(8):1019-32. doi: 10.1007/s10803-010-1125-5.

[11] Morel A, Peyroux E, Leleu A, Favre E, Franck N, Demily C. Overview of Social Cognitive Dysfunctions in Rare Developmental Syndromes With Psychiatric Phenotype. Front Pediatr. 2018;6:102. doi: 10.3389/fped.2018.00102.

[12] Laje G, Morse R, Richter W, Ball J, Pao M, Smith AC. Autism spectrum features in Smith-Magenis syndrome. *Am J Med Genet C Semin Med Genet*. 2010;154C(4):456-462. doi:10.1002/ajmg.c.30275.

[13] Richards C, Jones C, Groves L, Moss J, Oliver C. Prevalence of autism spectrum disorder phenomenology in genetic disorders: a systematic review and meta-analysis. Lancet Psychiatry. 2015;2(10):909-16. doi: 10.1016/S2215-0366(15)00376-4.

[14] Dykens EM, Lee E, Roof E. Prader-Willi syndrome and autism spectrum disorders: an evolving story. J Neurodev Disord. 2011;3(3):225-37. doi: 10.1007/s11689-011-9092-5.

[15] Veltman MW, Craig EE, Bolton PF. Autism spectrum disorders in Prader-Willi and Angelman syndromes: a systematic review. Psychiatr Genet. 2005;15(4):243-54. doi: 10.1097/00041444-200512000-00006.

[16] Cox DM, Butler MG. The 15q11.2 BP1-BP2 microdeletion syndrome: a review. Int J Mol Sci. 2015;16(2):4068-82. doi: 10.3390/ijms16024068.

[17] Kalsner L, Chamberlain SJ. Prader-Willi, Angelman, and 15q11-q13 Duplication Syndromes. Pediatr Clin North Am. 2015;62(3):587-606. doi: 10.1016/j.pcl.2015.03.004.

[18] Hartshorne TS. Behavioral phenotype. In: Hartshorne TS, Hefner MA, Davenport SLH, Thelin JW, editors. CHARGE Syndrome. San Diego: Plural Publishing Inc, 2011. P. 317-326.

[19] Hartshorne TS, Stratton KK, Brown D, Madhavan-Brown S, Schmittel MC. Behavior in CHARGE syndrome. Am J Med Genet C Semin Med Genet. 2017;175(4):431-438. doi: 10.1002/ajmg.c.31588.

[20] Hersh JH, Bloom AS, Weisskopf B. Childhood Autism in a female with Coffin Siris Syndrome. J Dev Behav Pediatr. 1982;3(4):249-52. doi: 10.1097/00004703-198212000-00016.

[21] Milutinovic L, Grujicic R, Mandic Maravic V, Joksic I, Ljubomirovic N, Pejovic Milovancevic M. Autism spectrum disorder and Coffin-Siris syndrome-Case report. Front Psychiatry. 2023;14:1199710. doi: 10.3389/fpsyt.2023.1199710.

[22] Baumgardner TL, Reiss AL, Freund LS, Abrams MT. Specification of the neurobehavioral phenotype in males with fragile X syndrome. Pediatrics. 1995;95(5):744-52.

[23] Hah M, Lotspeich LJ, Phillips JM, Torres AD, Cleveland SC, Hallmayer JF. Twins with KBG syndrome and autism. J Autism Dev Disord. 2009;39(12):1744-6. doi: 10.1007/s10803-009-0811-7.

[24] Ockeloen CW, Willemsen MH, de Munnik S, van Bon BW, de Leeuw N, Verrips A, Kant SG, Jones EA, Brunner HG, van Loon RL, Smeets EE, van Haelst MM, van Haaften G, Nordgren A, Malmgren H, Grigelioniene G, Vermeer S, Louro P, Ramos L, Maal TJ, van Heumen CC, Yntema HG, Carels CE, Kleefstra T. Further delineation of the KBG syndrome phenotype caused by ANKRD11 aberrations. Eur J Hum Genet. 2015;23(9):1176-85. doi: 10.1038/ejhg.2014.253. Erratum in: Eur J Hum Genet. 2015;23(9):1270.

[25] Skjei KL, Martin MM, Slavotinek AM. KBG syndrome: report of twins, neurological characteristics, and delineation of diagnostic criteria. Am J Med Genet A. 2007;143A(3):292-300. doi: 10.1002/ajmg.a.31597.

[26] Schmidt S, Nag HE, Hunn BS, Houge G, Hoxmark LB. A structured assessment of motor function and behavior in patients with Kleefstra syndrome. Eur J Med Genet. 2016;59(4):240-8. doi: 10.1016/j.ejmg.2016.01.004.

[27] Willemsen MH, Vulto-van Silfhout AT, Nillesen WM, Wissink-Lindhout WM, van Bokhoven H, Philip N, Berry-Kravis EM, Kini U, van Ravenswaaij-Arts CM, Delle Chiaie B, Innes AM, Houge G, Kosonen T, Cremer K, Fannemel M, Stray-Pedersen A, Reardon W, Ignatius J, Lachlan K, Mircher C, Helderman van den Enden PT, Mastebroek M, Cohn-Hokke PE, Yntema HG, Drunat S, Kleefstra T. Update on Kleefstra Syndrome. Mol Syndromol. 2012;2(3-5):202-212. doi: 10.1159/000335648.

[28] National Institutes of Health [Internet]. Kleefstra Syndrome. Available from: <https://www.ncbi.nlm.nih.gov/books/NBK47079/> Accessed 2 April 2024.

[29] Wingbermuehle E, Egger J, van der Burgt I, Verhoeven W. Neuropsychological and behavioral aspects of Noonan syndrome. Horm Res. 2009;72 Suppl 2:15-23. doi: 10.1159/000243774.

[30] Sena C, Iannello G, Skowronski AA, Dannheim K, Cheung L, Agrawal PB, Hirschhorn JN, Zeitler P, LeDuc CA, Stratigopoulos G, Thaker VV. Endocrine and behavioural features of Lowe syndrome and their potential molecular mechanisms. J Med Genet. 2022;59(12):1171-1178. doi: 10.1136/jmedgenet-2022-108490.

[31] Lane C, Milne E, Freeth M. Cognition and Behaviour in Sotos Syndrome: A Systematic Review. PLoS One. 2016;11(2):e0149189. doi: 10.1371/journal.pone.0149189.
